# The Mediterranean Red Alga *Asparagopsis*: A Source of Compounds against *Leishmania*

**DOI:** 10.3390/md7030361

**Published:** 2009-08-11

**Authors:** Giuseppa Genovese, Laura Tedone, Mark T. Hamann, Marina Morabito

**Affiliations:** 1 Department of Life Sciences "M. Malpighi"-Botany, University of Messina, Salita Sperone, 31, 98166 Messina, Italy; E-Mails:laura.tedone@gmail.com (L.T.);morabitom@unime.it (M.M.); 2 School of Pharmacy, University of Mississippi, 407 Faser Hall, University MS 38677, USA; E-Mail:mthamann@olemiss.edu

**Keywords:** antiprotozoal products, Asparagopsis armata, Asparagopsis taxiformis, halogenated metabolites, Leishmania, marine natural products

## Abstract

Crude extracts and column fractions from the red algae *Asparagopsis taxiformis* and *A. armata* from the Strait of Messina (Italy) were screened for the production of antimicrobial compounds. Extracts from both species revealed remarkable antiprotozoal activity against *Leishmania*, revealing such algae as a great source of natural antiprotozoal products.

## 1. Introduction

The interest in marine organisms as a potential and promising source of pharmaceutical agents has increased during the last years [1–4]. To date, many chemically unique compounds of marine origin with various biological activities have been isolated, and some of them are under investigation and are being used to develop new pharmaceuticals [5].

Some red algae were reported to produce chemicals that have potent biological effects [6,7]. Numerous natural products, including halogenated compounds, like haloforms, methanes, ketones, acetates and acrylates, were described just from the genus *Asparagopsis* [8,9].

This genus includes tropical-subtropical red seaweeds, with diplohaplontic life cycle and an heteromorphic tetrasporophyte known as the ‘*Falkenbergia’* stage. To date the genus is represented in the Mediterranean Sea by two species, *A. armata* and *A. taxiformis. A. armata*, a temperate distributed species, is considered a Lessepsian immigrant, first reported from the Algerian coasts in 1923 [10]. *A. taxiformis*, a tropical to warm temperate species, is considered a pre-Lessepsian immigrant or native in the eastern Mediterranean [11], since the first record in the Mediterranean Sea was given in Egypt [12]. Both taxa exhibit a strong invasive behaviour and are included in the list of the “Worst invasive alien species threatening biodiversity in Europe” (EEA 2007) and also in the list of the 100 “Worst Invasives in the Mediterranean Sea” [13].

*A. taxiformis* and *A. armata*, as well as other species of the family Bonnemaisoniaceae are well known as sources of halogenated compounds [8,14] with strong antifungal and antibiotic activity [8,15]. Dichloromethane extracts obtained from *A. armata* exhibited a strong activity against fish pathogenic bacteria [16].

In general, the production of biologically-active metabolites is inherently linked to an ability to partition compounds into specialised storage structures in order to avoid autotoxicity [17].

Members of the Bonnemaisoniaceae form specialized cells [18–20] typically known as vesicle or gland cells [21]. In the tetrasporophyte of *A. armata*, the halogenated metabolites accumulate as a refractile inclusion inside specialized gland cells and this inclusion is no longer produced when the alga is cultured without bromine [21].

The pungent aroma of these algae is due to an essential oil that is composed mainly of bromoform with smaller amounts of other bromine, chlorine, and iodine-containing methane, ethane, ethanol, acetaldehydes, acetones, 2-acetoxypropanes, propenes, epoxypropanes, acroleins and butenones [14]. The halogenated compounds from *Asparagopsis* have a wide range of volatility and solubility and, hence, no single method of extraction and isolation can be considered entirely satisfactory [8].

In the present paper we present data on the production of antimicrobial halogenated compounds on crude extracts and column fractions from *A. taxiformis* and *A. armata* against *Leishmania*.

## 2. Results and Discussion

Three different solvents with increasing polarity were used for the extraction of freeze-dried seaweed powder. Our observations of the effects of extraction method on bioactivity revealed that the active compounds are probably non polar to moderate polar, since the highest activity was observed in ethanol crude extracts fractioned with hexane:ethyl acetate and ethyl acetate. A series of small molecular volatile halogenated compounds (halomethanes, haloethers, haloacetals) are described as responsible for the antimicrobial action of *A. armata* [8].

Hexane and dichloromethane crude extracts of *A. taxiformis* exhibited a strong inhibition of *Leishmania.* IC_50_ (half maximal inhibitory concentration) were 17.00 μg/mL and 16.00 μg/mL, for both extracts, respectively, and IC_90_ (90% inhibitory concentration) were 33.00 μg/mL and 32.00 μg/mL, for both extracts, respectively (Table 1).

Hexane and dichloromethane crude extracts of *A. armata* also showed a remarkable inhibition, however, both IC_50_ and IC_90_ values were low (over 40.00 μg/mL) at the same experimental conditions (Table 1).

The active fractions obtained from ethanol crude extracts of *A. taxiformis* were eluted with hexane-ethyl acetate and ethyl acetate. IC_50_ values were 14.00 μg/mL and 20.00 μg/mL, for both fractions, respectively, and IC_90_ were 32.00 μg/mL and 34.00 μg/mL, for both fractions, respectively (Table 1).

The same moderate polar fractions from *A. armata* resulted active with IC_50_ of 10.00 and 19.00 μg/mL, IC_90_ 30.00 and 32.00 μg/mL under the same experimental conditions (Table 1).

Pentamidine and amphotericin B were tested as control drugs. Two different inhibition assays were performed. IC_50_ values ranged from 0.9 to 1.0 mg/mL and IC_90_s ranged from 1.9 to 4.0 mg/mL for pentamidine, while IC_50_s ranged from 0.18 to 0.19 mg/mL and the IC_90_ was 0.32 mg/mL for amphotericin B (Table 2).

Leishmaniasis is a disease caused by the protozoa of the *Leishmania* species and it has a worldwide distribution, especially in many tropical and sub-tropical countries. It affects as many as 12 million people worldwide, with 1.5–2 million new cases each year. There is increasing awareness that drug treatment can be complicated by variation in the sensitivity of *Leishmania* species to drugs, variation in pharmacokinetics, and variation in drug-host immune response interaction [26,27].

The LC-MS analysis of the column fraction in ethyl acetate from ethanol crude extracts of *A. taxiformis* revealed two peaks of nearly the same intensity at *m/z* 303.1 and 305.1 [M+H], which indicates presence of one bromine atom. Due to the small quantities of extracts and fractions, further characterization of this compound was not possible. The presence of a small molecular weight brominated molecule in the active fraction confirms that the lipophilic halogenated compounds are truly the metabolites responsible for potent antimicrobial activity of this extract.

## 3. Experimental Section

Plants of *A. taxiformis* and *A. armata* were collected from the Strait of Messina (Italy), respectively at Torre Faro, Messina and Villa San Giovanni, Reggio Calabria in May 2008. Fresh plants were washed in sterile sea water and manually cleaned of epiphytes. Lyophilized and powdered plants of *A. taxiformis* and *A. armata* (dry weights: 75 g for each species) were extracted using different organic solvents with increasing polarity (hexane, dichloromethane and ethanol) at room temperature. Extracts were dried with a Rotavapor® at low temperature (35 °C) to prevent volatile compounds from evaporation.

*In vitro* antimicrobial susceptibility assays were performed on *Leishmania donovani* promastigotes cultures (2 × 10^6^ cell/mL). A transgenic cell line of *L. donovani* promastigotes showing stable expression of luciferase was used as the test organism. The plates were incubated at 26 °C for 72 h, and growth of *Leishmania* promastigotes was determined by the Alamar blue assay [28]. Pentamidine and amphotericine B were tested as the standard antileishmanial agents. Microbiological assays were performed at the Microbiology laboratory of National Center For Natural Products Research of the University of Mississippi.

The hexane and dichloromethane extracts were not further fractionated because of limited amount of materials. Ethanol extracts of *A. taxiformis* and *A. armata* were submitted to fractionation using Si gel vacuum liquid chromatography eluted in order with hexane, hexane-ethyl acetate (1:1), ethyl acetate, ethyl acetate- methanol (1:1), methanol, water. Fractions were tested in antimicrobial assays.

Fractionation and isolation of compounds were further performed using HPLC, with a normal phase Silica gel column (10 mm) as stationary phase and gradient of two solvents, hexane and isopropanol, as mobile phase. Each fraction was dried in vacuum and ^1^H-NMR spectra in CDCl_3_ was recorded on a Bruker BioSpin instrument operating at 400 MHz. LC-MS analysis for each sample was carried out with a micrOTOF ESI-TOF MS.

## 4. Conclusions

Red algae of the genus *Asparagopsis* are well known as sources of halogenated compounds with strong antifungal and antibacterial activity [8,14–16], but, as far as we know, there are no published data on their activity against any protozoa. According to our results, *A. armata* and *A. taxiformis* revealed high potential as source of natural products with antiprotozoal activity *in vitro.* This first report of the effectiveness of *Asparagopsis* against *Leishmania* represents a challenge to encourage explorative research on such topic. *Asparagopsis* species merit further studies both with the aim of isolating their active metabolites on larger scale and for assaying culture methods for supplying algal biomass for industry.

## Figures and Tables

**Table 1:** Data of IC_50_ and IC_90_ (μg/mL) of crude extracts and fractions of *A. armata* and *A. taxiformis*. HEX: hexane; DCM: dichloromethane; EtOH: ethanol; EtOAc: ethyl acetate; MeOH: methanol.

Species	Crude extract/Fraction	IC_50_ (μg/mL)	IC_90_ (μg/mL)
*A. armata*	HEX	>40	>40
*A. armata*	DCM	>40	>40
*A. armata*	EtOH-Hex:EtOAc	10	30
*A. armata*	EtOH-EtOAc	19	32
*A. armata*	EtOH-EtOAc:MeOH	Inactive	Inactive
*A. armata*	EtOH-MeOH	Inactive	Inactive
*A. armata*	EtOH-H_2_O	Inactive	Inactive
*A. taxiformis*	HEX	17	33
*A. taxiformis*	DCM	16	32
*A. taxiformis*	EtOH-Hex:EtOAc	14	32
*A. taxiformis*	EtOH-EtOAc	20	34
*A. taxiformis*	EtOH-EtOAc:MeOH	Inactive	Inactive
*A. taxiformis*	EtOH-MeOH	Inactive	Inactive
*A. taxiformis*	EtOH-H_2_O	Inactive	Inactive

**Table 2:** Data of IC_50_ and IC_90_ (μg/mL) of tested control drugs.

Control drug	IC_50_ (mg/mL)	IC_90_ (mg/mL)
Pentamidine	from 0.9 to 1	from 1.9 to 4
Amphotericin B	from 0.18 to 0.19	0.32

## References

[1] Lindequist U Schweder T Rehm HJ Reed G Biotechnology Marine Biotechnology Wiley-VCH Weinheim, Germany 2001 10 441 484

[2] Mayer AMS Hamann MT Marine pharmacology in 2001–2002: Marine compounds with anthelmintic, antibacterial, anticoagulant, antidiabetic, antifungal, anti-inflammatory, antimalarial, antiplatelet, antiprotozoal, antituberculosis, and antiviral activities; affecting the cardiovascular, immune and nervous systems and other miscellaneous mechanisms of action Comp Biochem Physiol C: Toxicol Pharmacol 2005 140 265 286 15919242 10.1016/j.cca.2005.04.004 PMC4928201

[3] Newman DJ Cragg GM Snader KM Natural products as sources of new drugs over the period 1981–2002 J Nat Prod 2003 66 1022 1037 12880330 10.1021/np030096l

[4] Blunt JW Copp BR Hu W-P Munro MHG Northcote PT Prinsep MR Marine natural products Nat Prod Rep 2008 25 35 94 18250897 10.1039/b701534h

[5] Tüney Ü Çadirci BH Ünal D Sukatar A Antimicrobial activities of the extracts of marine algae from the coast of Urla (Üzmir, Turkey) Turk J Biol 2006 30 171 175

[6] Fenical W Halogenation in the Rhodophyta-a review J Phycol 1975 11 245 259

[7] Fenical W Natural products chemistry in the marine environment Science 1982 215 923 928 17821350 10.1126/science.215.4535.923

[8] McConnell O Fenical W Halogen chemistry of the red alga *Asparagopsis* Phytochemistry 1977 16 367 374

[9] Woolard FX Moore RE Roller PP Halogenated acetic and acrylic acids from the red alga *Asparagopsis taxiformis* Phytochemistry 1979 18 617 620

[10] Feldmann J Feldmann G Recherches sur les Bonnemaisoniacés et leur alternance de générations Ann Sci Nat Bot 1942 11 75 175

[11] Andreakis N Procaccini G Kooistra W *Asparagopsis taxiformis* and *Asparagopsis armata* (Bonnemaisoniales, Rhodophyta): Genetic and morphological identification of Mediterranean populations Eur J Phycol 2004 39 273 283

[12] Delile AR Anon Florae Aegyptiacae illustratio Description de l’Egypte ou recueil des observations et des recherches qui ont été faites en Egypte pendant l’expédition de l’armée française (1798–1801) Histoire naturelle Paris: France 1813 2 49 82

[13] Streftaris NS Zenetos A Alien marine species in the Mediterranean - the 100 ‘Worst Invasives’ and their impact Mediterr Mar Sci 2006 7 87 118

[14] Burreson BJ Moore RE Roller PP Volatile halogen compounds in the alga *Asparagopsis taxiformis* (Rhodophyta) J Agric Food Chem 1976 24 856 861

[15] Salvador N Garreta AG Lavelli L Ribera MA Antimicrobial activity of Iberian macroalgae Scientia Marina 2007 71 101 113

[16] Bansemir A Blume M Schröder S Lindequist U Screening of cultivated seaweeds for antibacterial activity against fish pathogenic bacteria Aquaculture 2006 252 79 84

[17] McKey D Rosenthal GA Janzen DH The distribution of secondary compounds within plant Herbivores: Their Interactonion with Secondary Plant Metabolites Academic Press San Diego, CA, USA 1979 56 133

[18] Wolk CP Role of bromine in the formation of the refractile inclusions of the vesicle cells of the Bonnemaisoniaceae (Rhodophyta) Planta 1968 78 371 375 10.1007/BF00387095 24522768

[19] Young DN Comparative Fine Structure and Histochemistry of Vesiculate Cells in Selected Red Algae Dissertation University of California Berkeley, USA 1977

[20] Womersley HBS The marine benthic flora of southern Australia. Part IIIB Australian Biological Resurces Study Canberra, Australian 1996 392

[21] Paul NA Cole L de Nys R Steinberg PD Ultrastructure of the gland cells of the red alga *Asparagopsis armata* (Bonnemaisoniaceae) J Phycol 2006 42 637 645

[22] Knight FR Mackenzie DW Evans BG Porter K Barrett NJ White GC Increasing incidence of Cryptococcosis in the United Kingdom J Infect 1993 27 185 191 8228302 10.1016/0163-4453(93)94863-7

[23] Huston SM Mody CH Cryptococcosis: An emerging respiratory mycosis Clin Chest Med 2009 30 253 264 19375632 10.1016/j.ccm.2009.02.006

[24] Kauffman CA Goldman L Ausiello D Cryptococcosis Cecil Medicine23rd ed Saunders Elsevier Philadelphia, PA, USA 2007

[25] Kirandeep K Meenakshi J Tarandeep K Rahul J Antimalarials from nature Bioorg Med Chem 2009 17 3229 3256 19299148 10.1016/j.bmc.2009.02.050

[26] Croft SL Sundar S Fairlamb AH Drug resistance in Leishmaniasis Clin Microbiol Rev 2006 19 111 126 16418526 10.1128/CMR.19.1.111-126.2006 PMC1360270

[27] Myler PJ Fasel N Leishmania: After the Genome Caister Academic Press Wymondham, UK 2008

[28] Mikus J Steverding D A simple colorimetric method to screen drug cytotoxicity against *Leishmania* using the dye Alamar Blue® Parasitol Int 2000 48 265 269 11227767 10.1016/s1383-5769(99)00020-3

